# Multi-Focus Image Fusion Based on Hessian Matrix Decomposition and Salient Difference Focus Detection

**DOI:** 10.3390/e24111527

**Published:** 2022-10-25

**Authors:** Xilai Li, Xiaopan Wang, Xiaoqi Cheng, Haishu Tan, Xiaosong Li

**Affiliations:** 1 Guangdong-Hong Kong-Macao Joint Laboratory for Intelligent Micro-Nano Optoelectronic Technology, School of Physics and Optoelectronic Engineering, Foshan University, Foshan 528225, China; 2 Guangdong Kegu Laboratory Technology Research Institute, Foshan 528225, China; 3 Ji Hua Laboratory, Foshan 528000, China

**Keywords:** multi-focus image fusion, Hessian matrix decomposition, salient difference focus detection

## Abstract

Multi-focus image fusion integrates images from multiple focus regions of the same scene in focus to produce a fully focused image. However, the accurate retention of the focused pixels to the fusion result remains a major challenge. This study proposes a multi-focus image fusion algorithm based on Hessian matrix decomposition and salient difference focus detection, which can effectively retain the sharp pixels in the focus region of a source image. First, the source image was decomposed using a Hessian matrix to obtain the feature map containing the structural information. A focus difference analysis scheme based on the improved sum of a modified Laplacian was designed to effectively determine the focusing information at the corresponding positions of the structural feature map and source image. In the process of the decision-map optimization, considering the variability of image size, an adaptive multiscale consistency verification algorithm was designed, which helped the final fused image to effectively retain the focusing information of the source image. Experimental results showed that our method performed better than some state-of-the-art methods in both subjective and quantitative evaluation.

## 1. Introduction

The limited depth of field of a camera causes it to only focus on one area of a scene, which makes it impossible for it to focus on all objects simultaneously. Multi-focus image fusion (MFIF) [1] aims to integrate multiple images of the same scene captured at different focal length settings into a fused all-in-focus image, which can be considered a collection of optimally focused pixels extracted from a set of source images. Fused images can provide a more comprehensive, objective, and reasonable interpretation of a scene than single-source images. Currently, MFIF is widely used in various fields, including microscopic imaging, weather phenomenon detection, intelligent surveillance, and so on. MFIF methods generally focus on the pixel level and can be approximately classified into deep-learning-, transform domain-, and spatial domain-based methods.

Recently, deep-learning techniques have become popular in computer vision [2], and several deep-learning-based image fusion methods have emerged [3,4,5,6]. Convolutional neural network (CNN) and adversarial generative network (GAN)-based fusion frameworks are two commercial multi-focus image fusion frameworks. Liu et al. [7] first introduced CNNs into the multi-focus fusion task. This scheme generates a focus decision map by learning a binary classifier to determine whether each pixel is focused and optimizes this map using postprocessing methods, such as consistency verification, to improve the quality of the fusion results.

Amin-Naji et al. [6] proposed an ensemble-learning-based method that directly generates the final decision map by combining the decision maps of different models to reduce the postprocessing steps and improve the computational efficiency of the fusion algorithm. Unlike CNN methods, decision graph-based GAN methods use generators to learn the mapping relationship from source images to decision graphs and generate fusion results, which also perform better in the field of multi-focus image fusion. Zhang et al. [4] proposed an unsupervised GAN with adaptive and gradient joint constraints to fuse multi-focus images, which effectively preserves their sharpness and textural details. Although deep-learning-based methods can be rapidly developed in multi-focus image fusion, and in turn, advance the technology, they also have the following drawbacks [8]: (1) Most deep-learning-based methods use starkly different key points for different image task settings to achieve good fusion performance. Different processing measures are designed for different tasks, which results in insufficient generalizability of such methods. (2) Most supervised methods use synthetic datasets. However, these training data are not consistent with real multi-focus images. Consequently, they cannot efficiently process real multi-focus images.

Transform-domain-based methods involve three main steps: decomposition, fusion, and reconstruction. The fusion process also includes the design of fusion rules, whereas decomposition and reconstruction are performed using a certain decomposition tool of the transform domain. Examples of classical algorithms are contrast pyramid [9], Laplace pyramid [10], and discrete wavelet transform [11]. Subsequently, multiscale geometric analysis tools with higher directional sensitivity than the wavelet transform were developed, such as nonsubsampled contourlet transform (NSCT) [12] and nonsubsampled shearlet transform [13].

Recently, the method based on the transform domain has been actively used because of its similarity to the human visual system, which is from close to fine. To improve the performance of the multi-focus image fusion method based on the transform domain, Li et al. [12] combined residual removal with NSCT to leverage the visual effect characteristics of the transform domain at the focus edges. Sparse representation (SR)-based methods fuse in the sparse domain and include image decomposition and reconstruction processes. Therefore, SR [14] can serve as an important branch of transform domain technology. The core idea of SR is that image signals can be represented as a linear combination of a “few” atoms from a pre-learned dictionary. SR-based methods usually employ a sliding window technique to achieve an approximately translational invariant fusion process.

Yang et al. [15] first introduced SR in multi-focus image fusion. However, the performance of model fusion did not achieve satisfactory results owing to the use of a fixed-basis-based dictionary. Zhang et al. [16] developed an image fusion algorithm based on analytic–synthetic dictionary pairs, which effectively combines the advantages of analysis and synthesis SR to improve the fusion performance of SR. With the development of SR, convolutional SR [17] has been used to solve the block-effect problem that frequently occurs with SR-based methods. In addition, SR methods based on low-rank sparse decomposition and dictionary learning have emerged [18,19], and these methods generally achieve image fusion and quality enhancement simultaneously.

Although the transform domain-based methods have superior performance and powerful generalizability in image fusion, they also have three considerable drawbacks: (1) Most multiscale transformation methods are inefficient in handling multilayer and multidirectional decomposition, as details of the source image can be lost during decomposition and reconstruction. (2) The sparse coding methods based on SR are computationally complex and taxing, which is not conducive to practical applications. (3) Inadequate consideration of spatial consistency in the fusion process can cause the loss of spatial information, resulting in brightness or color distortion [20].

Spatial domain-based methods directly consider the intensity information of pixels on the source image and can retain more spatial information of the source image compared to the transform domain methods. Such methods can be broadly classified as pixel-, block-, and area-based methods. The simplest pixel-based method directly averages all pixels on the source image. However, this method is highly sensitive to noise and tends to lose detail as the contrast decreases. Several block- and region-based methods have been developed recently [21,22,23,24] to solve this problem and more comprehensively consider the spatial information of the source image.

In the block-based algorithm, the image is first decomposed into equal-size image blocks, and then the focus measurement (FM) is used to determine the focus blocks. However, the approach of choosing an appropriate block size is a major challenge. Larger blocks may contain pixels in both focused and out-of-focus regions, while smaller blocks are not conducive to FM. To address these limitations, a previous study [23] used quadtree decomposition in multi-focus image fusion.

Region-based algorithms [25] generally segment the source image into different regions before fusion. Although they can avoid the block effect to a certain extent, when a region is partially segmented, it will generate erroneous segmentation results and affect the quality of fusion results. Regardless of the type of algorithm, block- or area-based, the goal is to determine the focus characteristics of each pixel in the most appropriate way. Therefore, FM plays a critical role as a tool in determining whether a pixel is in or out of focus. It is highly dependent on the edge details and textural information of the image, which may yield suboptimal results if FM is performed directly on the source image.

In addition, FM’s judgment of the pixel focus characteristics can be incorrect due to the diversity and complexity of images, which usually occurs in the boundary between the focused and out-of-focus regions of the image. This also leads to artifacts at the boundaries of the fusion results of several sophisticated algorithms. To accurately determine the focusing characteristics of pixels, this study proposes a multi-focus image fusion method.

First, the Hessian matrix is introduced to decompose the source image and obtain the Hessian-matrix feature map of the source image, which highlights the significant information. Meanwhile, to accurately determine the locations of the focused pixel points and avoid interference from out-of-focus pixels at the focus boundary, a focus detection method based on the salient difference analysis strategy is proposed. This method can effectively detect pixels with significant activity using the significance difference between pixels, so that the focus information of the source image can be effectively integrated into the fused image.

The contributions of this study are as follows:

1. A simple yet effective multi-focus image fusion method based on the Hessian-matrix with salient-difference focus detection is proposed.
2. A pixel salient-difference maximization and minimization analysis scheme is proposed to weaken the influence of pixels with similar activity levels at the focus boundary. It can effectively distinguish pixels in the focus and out-of-focus regions and produce high-quality focus decision maps.
3. An adaptive multiscale-consistency-verification scheme is designed in the postprocessing stage, which can adaptively optimize the initial decision maps of different sizes, solving the limitations caused by fixed parameters.

The remainder of this paper is organized as follows: Section 2 introduces the Hessian matrix. Section 3 details the proposed multi-focus image fusion algorithm based on the Hessian matrix and focus difference analysis. Section 4 presents the experimental results and comparative experiments. Finally, the conclusions of the study are presented in Section 5.

## 2. Related Works

### Hessian Matrix and Image Decomposing

In multi-focus images, the focused areas contain more significant information, such as edges, structures, and textures, than the blurred areas. Generally, significant information is detected in the source image before constructing the focus decision map. Most algorithms based on FM are more sensitive to this edge and detail information. Therefore, the method to effectively detect the detailed information within the focused region is the central problem of multi-focus image fusion research. Xiao et al. [26] proposed an image-decomposition strategy based on a multiscale Hessian matrix to make FM perform better and reduce image blurring and pseudo-edge problems. The feature map obtained after image decomposition by this matrix can clearly express the feature information of the source image and facilitate the implementation of focus detection.

Source images $f_{A}$ and $f_{B}$ are used as the input, for which the Hessian matrix is defined as follows:

(1) $$H\left(x,y,\sigma \right)=\left[\begin{array}{cc} L_{xx}\left(x,y,\sigma \right) & L_{xy}\left(x,y,\sigma \right)\\ L_{xy}\left(x,y,\sigma \right) & L_{yy}\left(x,y,\sigma \right) \end{array}\right]$$

where $L_{xx}\left(x,y,\sigma \right)$ is the convolution of the Gaussian second-order partial derivative $\partial^{2}/\partial x^{2}g\left(\sigma \right)$ with image f at the point (x,y), and it is the same with $L_{xy}\left(x,y,\sigma \right)$ and $L_{yy}\left(x,y,\sigma \right)$.

As a Hessian matrix can extract features with rotational invariance from images at multiple scales [27], the Hessian matrix can be improved to a multiscale Hessian matrix (MSH) weighted at different scales as follows:

(2) $$MSH\left(x,y\right)=\overset{n}{\underset{j=1}{\sum }}\varpi_{j}H\left(x,y,\sigma_{j}\right)$$

where *j* is the *j*-th scale, *n* is the number of scales, and $\varpi_{j}$ is the weight at scale *j*. Inspired by reference [25], in this study, we take the weights $\varpi_{1}=0.8$ for scale σ=0.4, and $\varpi_{2}=0.2$ for scale σ=1.2. Based on Equation (2), the feature image FIM(x,y) of source images can be extracted by setting the threshold λ as follows:

(3) $$FIM\left(x,y\right)=\{\begin{array}{c} 1,MSH\left(x,y\right)>\lambda \\ 0,MSH\left(x,y\right)\leq \lambda \end{array}$$

We set λ=0.0002 as the threshold for extracting the image features. For more information about the Hessian matrix, read [26].

## 3. Proposed Multi-Focus Image Fusion Method

### 3.1. Overview

**Figure 1:**
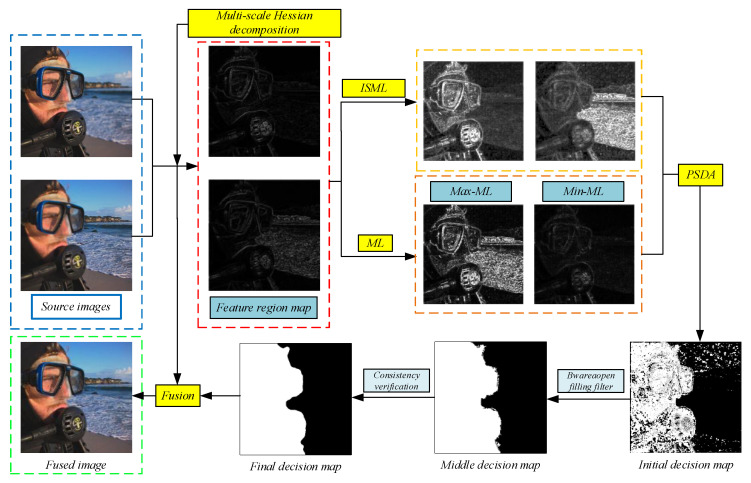
Flowchart of the proposed algorithm.

In this section, we present a detailed introduction to the multi-focus image fusion method based on a Hessian matrix with significant difference focus detection. Figure 1 depicts the general framework of fusion to better illustrate the general flow of the proposed algorithm. The proposed algorithm consists of four important stages: significant information representation, pixel salient-difference analysis, focused decision map optimization, and fusion result reconstruction. As shown in Figure 1, we decompose the source image in the first stage using a multiscale Hessian matrix to obtain the feature region map; subsequently, we use PSML and ML to process the feature region map to obtain four salient images reflecting different information. In the second stage, we process the four significant maps to obtain the initial focused decision map using the pixel salient-difference analysis scheme, and the detailed procedure is given in Section 3.3. In the third stage, the “*bwareaopen*” fill filter and the adaptive multiscale consistency verification algorithm are used to optimize the initial decision map and increase its accuracy in determining the focus properties of the pixels. In the final stage, the reconstruction of the fusion results is completed after the final decision map is obtained.

### 3.2. Significant Information Expression

The goal of multi-focus image fusion is to synthesize the focusing information from each source image; therefore, to obtain a fully focused image, the pixels being focused must be accurately determined. Typically, in multi-focus images, the focused area pixels tend to be more prominent than the out-of-focus area pixels. Therefore, we can first obtain the saliency map of the source image, and then determine the degree of pixel focus by judging the saliency of pixels. Thus, the saliency decision map corresponding to the source image can be obtained. The sum of the modified Laplacian (*SML*) is an effective tool for representing the significant information in images. The mathematical expression of the *ML* is as follows:

(4) $$\nabla_{ML}^{2}f\left(x,y\right)=\left|2f\left(x,y\right)-f\left(x-step,y\right)-f\left(x+step,y\right)\right|\;\;\;\;\;\;\;\;\;\;\;\;\;\;\;\;\;\;\;\;\;\;\;\;\;\;\;\;\;\;\;\;\;+\left|2f\left(x,y\right)-f\left(x,y-step\right)-f\left(x,y+step\right)\right|$$

where step denotes the step size, using variable spacing (step) between pixels to calculate the *ML* and thus adapt to changes in texture size, which can usually be set to 1. The *SML* is defined as follows:

(5) $$SML\left(f\left(x,y\right),\alpha \right)=\sum_{i=x-\alpha }^{i=x+\alpha }\sum_{j=y-\alpha }^{j=y+\alpha }\nabla_{ML}^{2}f\left(i,j\right)\;for\;\nabla_{ML}^{2}f\left(x,y\right)\geq L$$

where α is the radius of the window, L is the threshold set to 0, and α is set to 3.

Traditional *ML* only considers the pixels around the central one in both horizontal and vertical directions. Kong et al. [28] improved the traditional *ML* by considering the other four pixels on the diagonal that contain critical information. The improved *ML* expression is as follows:

(6) $$\nabla_{ML}^{2}f\left(x,y\right)=\left|2f\left(x,y\right)-f\left(x-step,y\right)-f\left(x+step,y\right)\right|+|2f\left(x,y\right)-\;f\left(x,y-step\right)-f\left(x,y+step\right)|+|2f\left(x,y\right)-f\left(x-step,y-step\right)-\;f\left(x+step,y+step\right)|+|2f\left(x,y\right)-f\left(x-step,y+step\right)-f(x+step,y-\;step)|$$

So the improved *SML* (*ISML*) can be expressed as follows:

(7) $$ISML\left(f\left(x,y\right),\alpha \right)=\sum_{i=x-\alpha }^{i=x+\alpha }\sum_{j=y-\alpha }^{j=y+\alpha }\nabla_{ML}^{2}f\left(i,j\right)\;for\;\nabla_{ML}^{2}f\left(i,j\right)\geq L$$

The *ISML* value of each pixel of the feature region map *FIM* obtained by Equation (3) is expressed as follows:

(8) $$M_{A}=ISML\left(FIM_{A}\left(x,y\right),\alpha \right)$$

(9) $$M_{B}=ISML\left(FIM_{B}\left(x,y\right),\alpha \right)$$

where $FIM_{A}$ and $FIM_{B}$ are the feature region maps obtained by using the Hessian matrix to decompose the source image $f_{A}$ and $f_{B}$, respectively. Furthermore, $M_{A}$ and $M_{B}$ are the saliency maps of $FIM_{A}$ and $FIM_{B}$, respectively.

### 3.3. Pixel Salient Difference Analysis (PSDA)

First, we calculate its modified Laplace value within a small window near the pixel origin (x,y).

(10) $$S_{A}\left(x,y\right)=\nabla_{ML}^{2}FIM_{A}\left(x,y\right)$$

(11) $$S_{B}\left(x,y\right)=\nabla_{ML}^{2}FIM_{B}\left(x,y\right)$$

To find the most and least salient pixel points in the source image, the maximum and minimum *ML* maps of the source image are found, respectively, and are mathematically expressed as follows:

(12) $$S_{max}\left(x,y\right)=max\left(S_{A}\left(x,y\right),S_{B}\left(x,y\right)\right)$$

(13) $$S_{min}\left(x,y\right)=min\left(S_{A}\left(x,y\right),S_{B}\left(x,y\right)\right)$$

where $S_{max}\left(x,y\right)$ and $S_{min}\left(x,y\right)$ are the maximum and minimum *ML* maps of all source images, respectively. $S_{max}$ can be approximated as a fully focused saliency map, while $S_{min}$ is a fully out-of-focus saliency map as *ML* can reflect the focusing information of the image, and the salient information of each position of $S_{A}\left(x,y\right)$ and $S_{B}\left(x,y\right)$ is contained in $S_{max}$. The difference salient map (*DSM*) between $S_{max}$ and $S_{min}$ can be calculated as follows:

(14) $$DSM=S_{max}-S_{min}$$

Meanwhile, the difference map (*DM*) between $M_{A}$ and $M_{B}$ can be calculated by:

(15) $$DM=M_{A}-M_{B}$$

**Figure 2:**
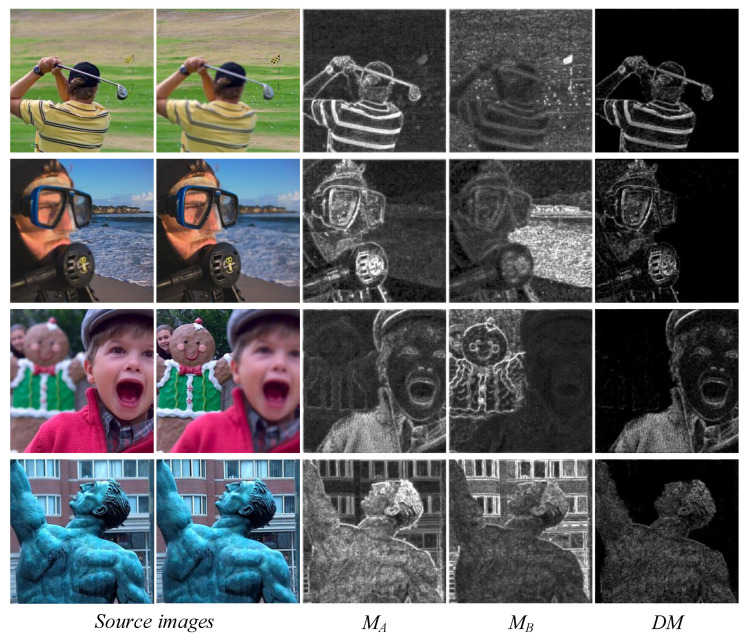
Source-image saliency maps and maximum–minimum difference maps.

**Figure 3:**
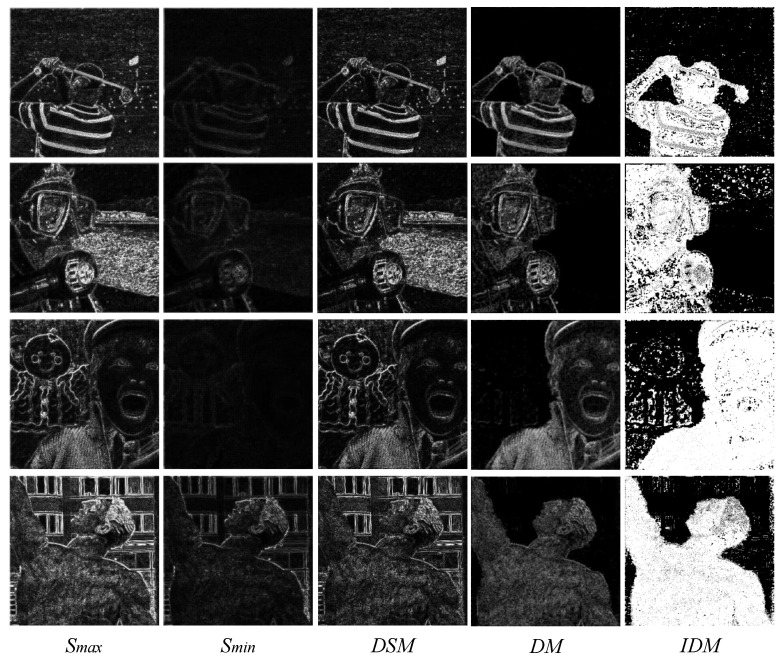
Intermediate process of pixel salient-difference analysis.

As observed in the *ISML* maps in Figure 2, the significant information in the source image can be effectively extracted using *ISML*. The *DM* is the difference map between two *ISML* maps. Only the focused region in the source image was retained in the *DM*, which can be clearly observed at the boundary between the focused and out-of-focus areas. Although the scheme can effectively detect the focused pixels, it also detects some false pixel information at the boundary of the salient map by *DM*. Figure 3 illustrates the intermediate process of pixel salient-difference analysis.

$S_{max}$ and $S_{min}$ in the figure represent the salient pixel information of fully focused and fully out-of-focus images, respectively, whereas *DSM* reflects the maximum difference of salient pixel information within the source image. Therefore, by comparing *DM* and *DSM*, we can judge the salience of pixels in the source image, and then reflect the focusing characteristics of those pixels. However, in the *DM*, $M_{A}$ and $M_{B}$ are the *ISML* maps of the source image, and the difference between them is not as evident as $S_{max}$ and $S_{min}$. We propose the following rules to comprehensively consider *DSM* and *DM*, and thus obtain the initial decision map (*IDM*):

(16) $$IDM=\{\begin{array}{l} 1,ifDM\geq \mu \times DSM\\ 0,otherwise \end{array}$$

where μ is a custom threshold.

### 3.4. Focused Decision Map Optimization

#### 3.4.1. Step 1—Small Area Removal Filter

**Figure 4:**
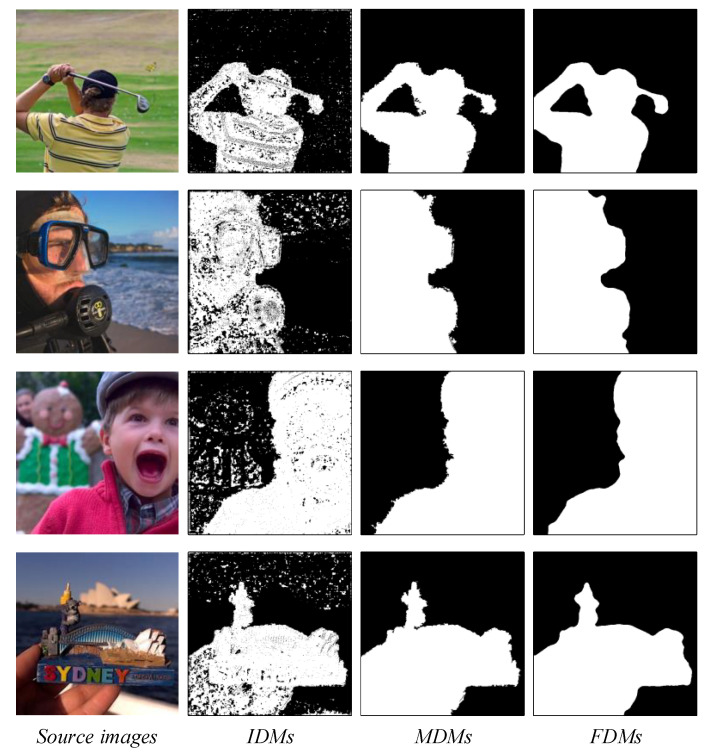
Optimization process of the decision maps.

Considering the inevitable presence of some wrongly selected pixel areas in the *IDM*. The *IDM*s in Figure 4 reveal a few small, isolated areas in the focus region, which consist of a few wrongly selected pixels. To solve this problem, we used the “*bwareaopen*” filter to eliminate the isolated areas or holes containing the erroneous pixels in the focus area.

(17) MDM=bwareaopen(IMD,S/45)

where *S* represents the area of the source image. Equation (17) was used to eliminate the isolated areas in the *IMD* smaller than *S/45* using the “*bwareaopen*” filter to obtain the middle decision map (*MDM*). Figure 4 illustrates the optimization process of the decision map. Figure 4 reveals that, compared with the *IMD*, the *MDM* can further correct the wrongly selected pixels in the decision map and improve the focus detection accuracy.

#### 3.4.2. Step 2—Adaptive multiscale consistency verification

Meanwhile, considering the consistency of the target, we used the consistency verification technique [29] to optimize the *MDM*. The traditional consistency verification technique only uses one window to determine whether the pixel is in the focus region.

(18) $$\hat{M}\left(x,y\right)=\{\begin{array}{l} 1,if\underset{\left(a,b\right)\in \delta }{\sum }MDM\left(x+a,y+b\right)\geq \delta /2\\ 0,otherwise \end{array}$$

where δ denotes a square field window centered at (x,y). However, this type of method is used with fixed window size and cannot effectively consider the values of the pixel under a varying size, which can easily cause pixel judgment errors in the boundary area between the focused and out-of-focus regions. Moreover, due to the diversity of images, a fixed window size may have different effects on different decision maps, and even lead to serious damage of the focus region in the decision map, introducing a large area of erroneous pixels. Therefore, the selection of window size is crucial for consistency verification. We proposed an adaptive multiscale consistency verification scheme to solve the problem effectively. We set up two windows to determine whether the pixels were focused, and the mathematical formula is expressed as follows:

(19) $$FDM\left(x,y\right)=\left\{ \begin{array}{l} 1,if\underset{\left(a,b\right)\in \delta_{A}}{\sum }MDM\left(x+a,y+b\right)-\underset{\left(i,j\right)\in \delta_{B}}{\sum }MDM\left(x+i,y+j\right)\geq \varphi^{2}/2\\ 0,otherwise \end{array}\right.$$

where $\delta_{A}$ and $\delta_{B}$ are two square domain windows of different sizes centered at (m,n), $\delta_{A}=T^{2}$, $\delta_{B}={\left(T-14\right)}^{2}$. *FDM* is the final decision map. Assuming that the size of the source image is M×N, $\varphi =log_{2}max\left(M,N\right)\times 14+3$. The final decision map in Figure 4 shows that the erroneously selected pixels at the boundary of the *MDM* have been removed, and the focus boundary has become smooth and complete.

### 3.5. Fusion Result Reconstruction

With the final decision map FDM obtained, the fusion result can be derived from the following equation:

(20) $$F\left(x,y\right)=\{\begin{array}{l} f_{A}\left(x,y\right),\;ifFDM\left(x,y\right)=1\\ f_{B}\left(x,y\right),\;otherise \end{array}$$

## 4. Experiments

### 4.1. Experimental Setup

The results were compared with 11 state-of-the-art fusion methods to verify the validity of the proposed method. Eight objective evaluation metrics were used for the quantitative analysis, and the specific experimental setup is explained below.

#### 4.1.1. Image Datasets

**Figure 5:**
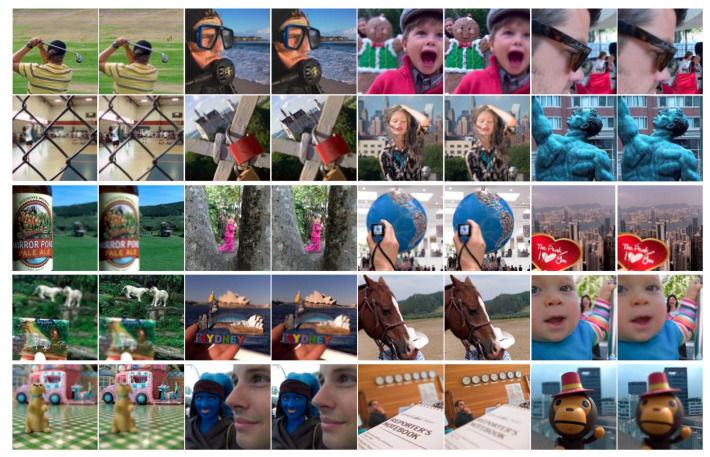
Color multi-focus image dataset. This dataset includes 20 sets of source images.

**Figure 6:**
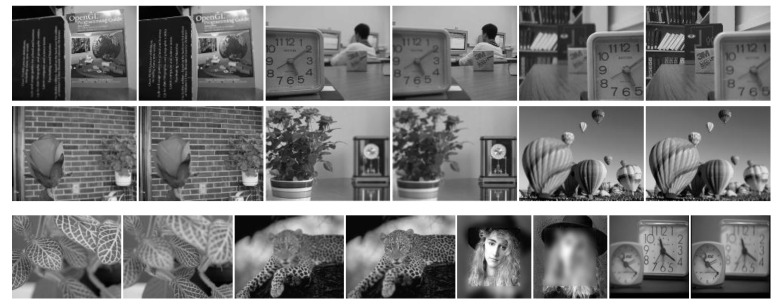
Grayscale multi-focus image dataset. This dataset contains 10 sets of source images.

We used two of the most popular publicly available datasets for testing, one of which is the “Lytro” dataset [30] of multi-focus color images (see Figure 5). The other is a classic grayscale multi-focus image dataset. [Online]. Available: http://imagefusion.org/ (accessed on 20 October 2022). (see Figure 6).

#### 4.1.2. Compared Methods

To verify the effectiveness and advancement of the proposed method, we compared 11 current state-of-the-art methods, as follows:

- Multi-focus image fusion based on NSCT and residual removal [12] (NSCT-RR).
- Multiscale weighted gradient-based fusion for multi-focus images [22] (MWGF).
- Multi-focus image fusion by Hessian matrix based decomposition [26] (HMD).
- Guided filter-based multi-focus image fusion through focus region detection [24] (GFDF).
- Analysis–synthesis dictionary-pair learning and patch saliency measure for image fusion [16] (YMY).
- Image fusion with convolutional sparse representation [17] (CSR).
- Ensemble of CNN for multi-focus image fusion [6] (ECNN).
- Towards reducing severe defocus spread effects for multi-focus image fusion via an optimization based strategy [5] (MFF-SSIM).
- MFF-GAN: An unsupervised generative adversarial network with adaptive and gradient joint constraints for multi-focus image fusion [4] (MFF-GAN).
- U2Fusion: A unified unsupervised image fusion network [31] (U2Fusion).
- IFCNN: A general image fusion framework based on convolutional neural network [32] (IFCNN).

Among them, NSCT-RR is a method of combining the spatial and transform domains. MWGF, HMD, and GFDF are spatial domain methods based on focus detection, YMY and CSR are methods based on SR, and ECNN, MFF-SSIM, MFF-GAN, U2Fusion and IFCNN are methods based on deep learning. The 11 methods cover various types of current multi-focus image fusion methods. In a sense, they also espouse the latest developments in the field. Therefore, the performance of the proposed method will be validated by comparison with them. The parameter settings of all 11 methods were identical to those in the respective published literature.

#### 4.1.3. Objective Evaluation Metrics

Objective evaluation has been a challenge in image fusion, and individual metrics cannot effectively reflect the full information of the fusion results. Therefore, to comprehensively and effectively evaluate the fusion results of different algorithms to compare the fusion performance, we used eight popular quantitative evaluation metrics for multi-focus image fusion:

- Normalized mutual information (Q_MI_) [33].
- Nonlinear correlation information entropy (Q_NCIE_) [34].
- Gradient-based fusion performance (Q_G_) [35].
- Image fusion metric based on a multiscale scheme (Q_M_) [36].
- Image fusion metric based on phase congruency (Q_P_) [37].
- Average gradient (AG) [38].
- Chen–Blum metric (Q_CB_) [39].
- Chen-Varshney metric (QCV) [40].

More specifically, Q_MI_ is a measure of the mutual information between the fused image and the source image, and Q_NCIE_ is used to measure the nonlinear correlation information entropy between the fusion result and the source image; it can be seen that these two metrics belong to the information theory-based metrics. Q_G_ is used to evaluate the amount of edge information, and Q_M_ is an image fusion metric based on a multi-scale scheme implemented using two-level Haar wavelets to measure the degree of edge preservation at different scale spaces. Q_P_ is a phase congruency-based metric since the moments contain corner and edge information of the image. Furthermore, the metric can be defined using the principal moments of the image phase coherence. AG represents the average gradient. A large value indicates that the fused image contains more gradient information, which means better fusion performance. Q_CB_ is constructed based on local saliency, global saliency, and similarity. It evaluates the fused image from the perspective of visual saliency. Both Q_CB_ and Q_CV_ belong to human perception-inspired fusion metrics. The above eight metrics can evaluate the fusion performance of different methods in a comprehensive way, which makes our experiments more convincing.

For Q_CV_, a smaller value indicates better performance, while a larger value means better performance for the rest of the seven metrics.

#### 4.1.4. Parametric Analysis

**Figure 7:**
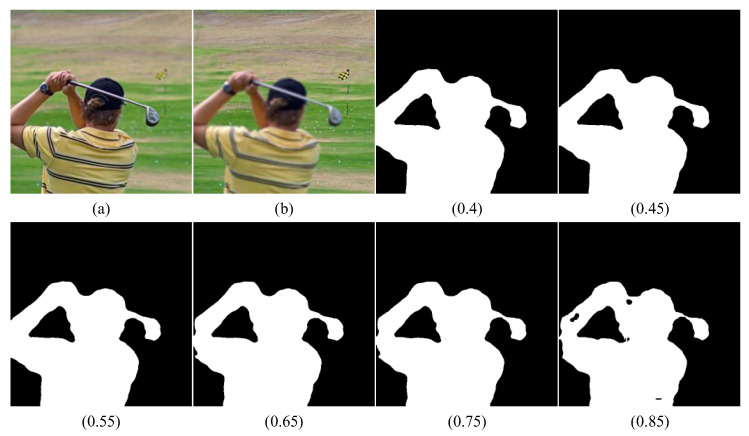
Results of the analysis of the parameter μ. (**a**,**b**) Two multi-focus source images, and the remainder are the final decision maps with different threshold values μ.

In the proposed method, two key parameters were to be analyzed: threshold μ in Equation (16) and threshold T in Equation (18) that controls the size of windows $\delta_{A}$ and $\delta_{B}$. To find the appropriate parameters, we selected a set of images from Lytro as the test images (Figure 7). First, we set the threshold T to 17 and varied μ. We observed that the boundary of the focus region became blurred when μ > 0.55, and a boundary discontinuity appeared at the site of the sportsman’s elbow. Meanwhile, when μ = 0.85, several small black holes appeared in the focus area, which caused the final fusion to contain the wrong pixel information. This scenario did not occur when μ = 0.4 or 0.45.

However, the golf club wielded by the sportsman can be observed. We observed that the boundary of the club becomes particularly thick when the value of μ is too small or significantly larger than the actual focused boundary. This results in residual artifacts at the border of the fused image. In summary, the best performance for image fusion was achieved when μ = 0.55. Hence, we set the threshold μ to 0.55.

μ was fixed at 0.55, and different thresholds T were separately set to find the best T value. Table 1 lists the average of the scores of six evaluation metrics for different parameters T on Lytro. The best scores are emboldened. As observed in the table, three metrics achieved the best score when T = 15 or 17. In terms of all the values, the performance of each metric was intermediate and high when T = 17. Hence, in this study, we set the threshold T to 17.

In summary, the values of the two key parameters can be set as (μ = 0.55, T = 17).

### 4.2. Subjective Analysis of the Fusion Results

**Figure 8:**
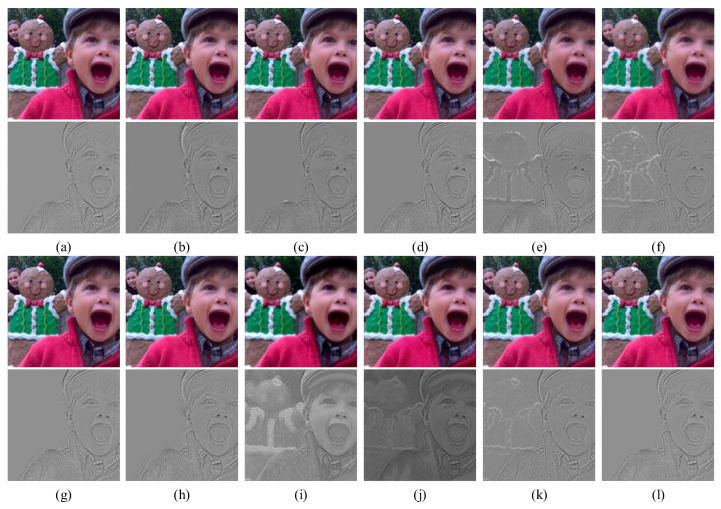
Fusion example 1: (**a**–**l**) Fusion results of NSCT-RR, MWGF, HMD, GFDF, YMY, CSR, ECNN, MFF-SSIM, MFF-GAN, U2Fusion, IFCNN and the proposed method, respectively.

The fusion results of the source image “children” in the Lytro dataset for different methods are displayed in Figure 8. Each fusion image was subtracted from the same source image to derive its own difference map. The cleaner the focused region in the difference map, the better the performance of the method. The figures demonstrate that all the methods obtained good fusion results. The difference map shows that the five methods YMY, CSR, MFF-GAN, U2Fusion and IFCNN still contained several pieces of background information in the out-of-focus region. Additionally, not all pixels of the fused image originated from the focused region on the source image, which resulted in the loss of some information and reduced the clarity of the image (Figure 8e,f,i–k).

Although the other methods did not show this effect, MWGF, HMD, GFDF, and ECNN also produced residual artifacts at the focus boundary, proving that none of them could better preserve the boundary between the focused and out-of-focus regions of the source image (Figure 8b–d,g). At the connection between the person’s hat and ear, the NSCT-RR method produced a discontinuous focus boundary, and the MFF-SSIM method produced blur at the boundary of the ear. The resulting fused image was inferior to the image obtained by the proposed method in several aspects (Figure 8a,h). In contrast, the focused region in the difference map of the proposed method was clean, and the focused boundary was continuous (Figure 8j). This indicates that most of the focused pixels in the source image were retained in the fusion result. In summary, the proposed method efficiently preserved the visual quality of the fusion results, judged the pixel focus characteristics, and led in terms of the overall performance compared to the 11 state-of-the-art methods.

**Figure 9:**
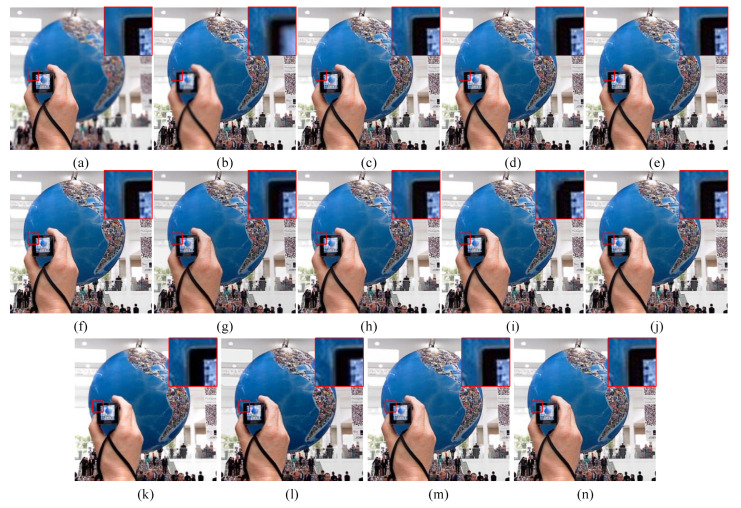
Fusion example 2: (**a**,**b**) The source images. (**c**–**n**) Fusion results of NSCT-RR, MWGF, HMD, GFDF, YMY, CSR, ECNN, MFF-SSIM, MFF-GAN, U2Fusion, IFCNN and the proposed method, respectively.

The fusion results of the source image “globe” in the Lytro dataset obtained by different methods are displayed in Figure 9. Figure 9a,b are two multi-focus source images, and Figure 9c–l highlight the fusion results obtained by different methods. For better comparison of the performances, local areas at the same location of each fused image were enlarged. First, as indicated in red, the magnified area of Figure 9, YMY, CSR, ECNN, MFF-GAN and U2Fusion produced images with blurred boundaries (Figure 9g–i,k,l).

Although the quality of fused images obtained by GFDF, MFF-SSIM and IFCNN methods was improved, the clarity was lower (Figure 9f,j,m). A closer examination of the edges of the person’s hand in the images under NSCT-RR, MWGF, and HMD revealed discontinuities (Figure 9c–e). Compared with the above methods, the proposed method produced the most continuous, complete, and clean focus boundary of the fused image (Figure 9l). Hence, the algorithm can effectively transfer the focusing information to the source image and effectively determine the focusing properties of the focusing boundary pixels.

**Figure 10:**
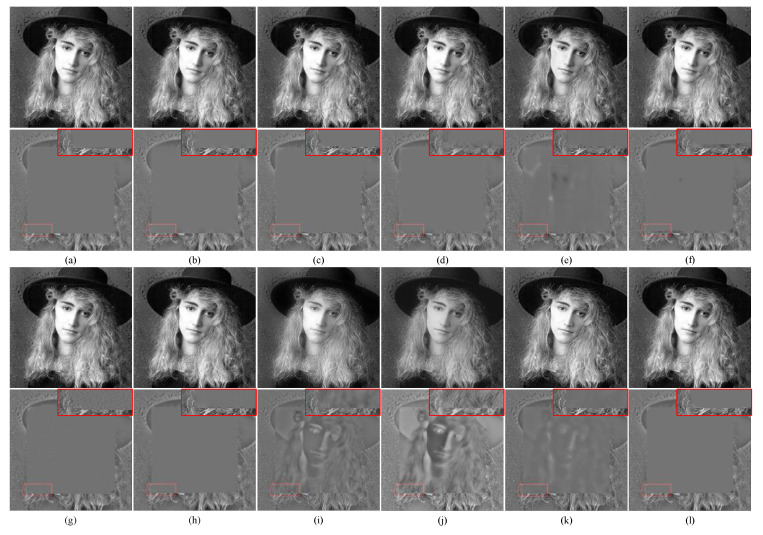
Fusion example 3: (**a**–**l**) Fusion results of NSCT-RR, MWGF, HMD, GFDF, YMY, CSR, ECNN, MFF-SSIM, MFF-GAN, U2Fusion, IFCNN and the proposed method, respectively.

Figure 10 illustrates the fusion results of the synthetic multi-focus source image “Woman in Hat” from the Grayscale dataset under different methods. Overall, all methods achieved acceptable fusion results. However, as observed in Figure 10e–k, the fusion results of YMY, CSR, ECNN, MFF-SSIM, MFF-GAN, U2Fusion and IFCNN methods lost some source image focus information and produced blurriness. For example, their difference maps revealed the presence of residual information in the out-of-focus regions.

The enlarged areas in Figure 10c–f confirmed that HMD, GFDF, YMY, and CSR could not accurately segment the focal region, and even produced incorrect segments at the boundaries. The MWGF method produced an evident artifact, which resulted in blurred edges of the fusion results (Figure 10b). Further, the NSCT-RR method effectively preserved the pixel information in the focused region on the source image (Figure 10a).

However, the performance of the proposed method for the retention of the focused boundary was superior to that of NSCT-RR, as it produced better visual effects at the boundary (Figure 10j). In summary, the proposed method distinguished the focused and out-of-focus regions more accurately than the other methods, and the resulting fusion results had better subjective visual performance.

### 4.3. Objective Analysis of the Fusion Results

In addition to the subjective visual comparison, we also objectively evaluated the 12 methods on the Lytro and Grayscale datasets. The top four scores are also indicated. As indicated in Table 2 and Table 3, the proposed method scored the best in terms of four indices, Q_MI_, Q_NICE_, Q_G_, and Q_CB_. It also scored the best in terms of energy information, detail information, and retention of human-eye visual effects. As indicated in Table 2, although the proposed algorithm did not have the best scores on the remaining four metrics, the scores of three metrics, Q_M_, Q_P_ and AG, were among the top three, and the score of metric Q_CV_ was above average. In addition, from Table 3, it can be found that the proposed algorithm had the top four scores in two metrics, QM and QP, and although it performed poorly in the remaining metrics AG and Q_CV_, the performance of the proposed algorithm was still in the leading position among all metrics.

Notably, although the GFDF method scored the best in terms of Q_P_ and Q_CV_, it exhibited mediocre performance in the other metrics. In addition, YMY, CSR, MFF-GAN, and U2Fusion performed relatively poorly. The quantitative evaluations of NSCT-RR, HMD, GFDF, and IFCNN algorithms placed them in the intermediate- to high-performance categories, and the scores of several indicators were among the top four scores. To summarize, the proposed algorithm outperformed the 11 methods in the quantitative evaluation. This conclusion is consistent with the subjective visual analysis in Section 4.2, which demonstrated the advantages of the proposed method in both subjective and objective evaluations compared with the state-of-the-art methods.

### 4.4. Robustness Test to Defocus Spread Effect (DSE)

**Figure 11:**
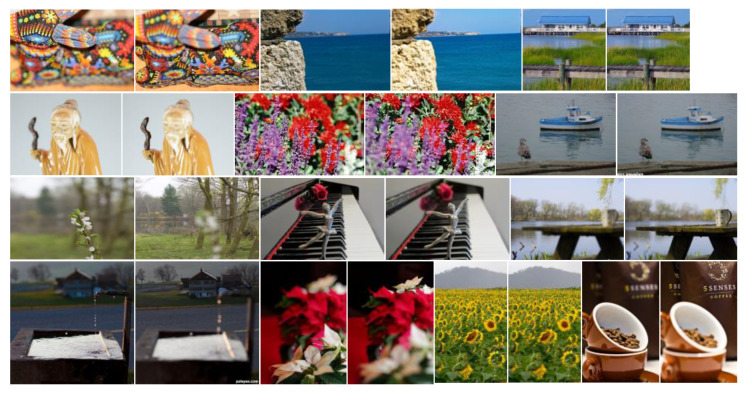
MFFW dataset. This dataset includes 13 sets of source images.

DSE is very important for MFIF, and many current state-of-the-art multi-focus image fusion algorithms ignore the existence of DSE in multi-focus images. Some objects that are not in focus are significantly enlarged in images that suffer from DSE, and they can cause the focus decision map to have pixel focus attributes misjudged to the extent that incorrect pixel information is introduced into the fusion results. We introduced the MFFW [41] dataset to verify the robustness of the proposed algorithm to DSE. The scenes inside this dataset are much more complex compared to the previous two datasets, and there is obvious DSE. It is a major challenge to achieve good fusion performance in MFFW dataset (Figure 11).

We performed a quantitative comparison between the proposed algorithm and the comparison method using test data from the MFFW dataset to demonstrate the robustness of the proposed algorithm to DSE. In addition, we changed the parameter μ in Equation (16) to 0.4 and the definition of the small area in Equation (17) to *S/30*. The parameters can be better adapted to the MFFW dataset by changing them to obtain good fusion results even in datasets that suffer from DSE.

For the quantitative comparison, we used eight evaluation metrics to score the different fusion results, and Table 4 lists the average of the scores of the various methods in the MFFW dataset.

Table 4 illustrates that the proposed algorithm achieved the best scores for four evaluation metrics and also achieved, Q_M_ and Q_P_. It is worth noting that NSCT-RR, MWGF, HMD, and GFDF also performed very well, achieving the top four scores in several metrics. Combining all the metrics, we can conclude that the proposed algorithm is robust to DSE and can still effectively retain the details and gradient information on the source images in the dataset suffering from DSE; further, the fusion performance is better than that of some state-of-the-art algorithms.

## 5. Conclusions

In this study, a multi-focus image fusion algorithm based on Hessian matrix decomposition and salient-difference focus detection is proposed. The method uses the multiscale Hessian matrix to extract the feature regions of the image to more comprehensively derive the salient information of the source image for FM. To accurately determine the focus characteristics of each pixel, a focal difference analysis scheme was proposed based on SML, which effectively improved the accuracy of judgment of the focusing characteristics of the pixels. Furthermore, considering that images of different sizes have different degrees of adaptability to the algorithm, an adaptive multiscale consistency verification algorithm that leverages the correlation between each pixel and its surrounding pixels was proposed to optimize the decision map. The method was compared with 11 state-of-the-art methods in an experiment, and all methods were tested on three multi-focus public datasets using eight popular metrics for quantitative analysis. The results showed that the proposed algorithm efficiently transferred the focusing information of the source images to the fusion results and outperformed some state-of-the-art algorithms in both subjective vision and objective evaluations. Further research should focus on thoroughly uncovering the impact of DSE on multi-focus image fusion and finding more efficient ways to solve the DSE problem.

## Figures and Tables

**Table 1:** Average quantitative evaluation in terms of different T on the Lytro dataset.

*T*	Q_MI_	Q_NICE_	Q_G_	Q_M_	Q_P_	AG	Q_CB_	Q_CV_
**15**	**1.1703**	**0.8447**	**0.7266**	2.9721	0.8501	6.9495	0.8085	20.6197
**17**	1.1675	0.8443	0.7261	2.9743	**0.8526**	**6.9556**	**0.8088**	17.3249
**19**	1.1656	0.8441	0.7253	**2.9747**	0.8519	6.9479	0.8077	16.5342
**21**	1.1646	0.8440	0.7244	2.9744	0.8499	6.9369	0.8078	**16.5145**
**23**	1.1637	0.8438	0.7232	0.9737	0.8478	6.9235	0.8076	16.7755

**Table 2:** Average experimental results of different MFIF methods on the Lytro dataset.

Method	Lytro Dataset							
	Q_MI_	Q_NICE_	Q_G_	Q_M_	Q_P_	AG	Q_CB_	Q_CV_
NSCT-RR [12]	1.1558(3)	0.8433(3)	0.7233(2)	2.9745(2)	0.8537(2)	6.9235	0.8077(2)	16.5356(3)
MWGF [22]	1.0800	0.8388	0.6962	2.9657	0.8299	6.8161	0.7883	19.0492
HMD [26]	1.1617(2)	0.8436(2)	0.7229(3)	**2.9749**	0.8517(4)	6.9344(4)	0.8070(3)	16.0576(2)
GFDF [24]	1.1433(4)	0.8424(4)	0.7169	2.9723(4)	**0.8540**	6.9003	0.8055(4)	**15.8881**
YMY [16]	0.9575	0.8308	0.6841	2.9450	0.8420	6.3056	0.7548	24.9025
CSR [17]	0.9271	0.8293	0.6267	2.9477	0.8317	6.2769	0.703	23.3846
ECNN [6]	1.1091	0.8403	0.7052	2.9698	0.831	6.9172	0.7981	16.5572(4)
MFF-SSIM [5]	1.1056	0.8402	0.7202(4)	2.9722	0.8509	**6.9664**	0.7994	17.1171
[4]	0.8549	0.8258	0.5934	2.9133	0.7698	6.9411(3)	0.6526	67.8443
U2Fusion [31]	0.7777	0.8224	0.5612	2.8988	0.7112	6.5318	0.6456	40.3313
IFCNN [32]	0.9336	0.8296	0.6673	2.9538	0.8273	6.8747	0.7296	19.956
Proposed	**1.1675**	**0.8443**	**0.7261**	2.9743(3)	0.8526(3)	6.9556(2)	**0.8088**	17.3249

**Table 3:** Average experimental results of different MFIF methods on the Grayscale dataset.

Methods	Grayscale Dataset							
	Q_MI_	Q_NICE_	Q_G_	Q_M_	Q_P_	AG	Q_CB_	Q_CV_
NSCT-RR [12]	1.2438(3)	0.8488(2)	0.7395(4)	2.9666(2)	0.8967(2)	8.0313	0.8188(3)	38.02
MWGF [22]	1.156	0.8433	0.7228	2.9511	0.8928	7.7235	0.8048	52.1807
HMD [26]	1.2497(2)	0.8487(3)	0.7423(2)	**2.967**	0.8958(3)	8.0079	0.8245(2)	37.2895(4)
GFDF [24]	1.2166(4)	0.8472(4)	0.7401(3)	2.9661(3)	**0.8983**	7.9566	0.8245(2)	**35.7523**
YMY [16]	1.0062	0.8336	0.6841	2.9494	0.8867	7.4494	0.774	37.4149
CSR [17]	1.0222	0.8347	0.6343	2.9581	0.8871	7.6903	0.7654	35.9312(2)
ECNN [6]	0.8323	0.8259	0.5408	2.9266	0.7732	8.1873(3)	0.7494	37.4752
MFF-SSIM [5]	0.995	0.8337	0.6633	2.9552	0.8787	8.087(4)	0.7934	37.2547(3)
MFF-GAN [4]	0.843	0.8264	0.5769	2.9055	0.8132	**8.7637**	0.7015	62.7001
U2Fusion [31]	0.7634	0.8232	0.5525	2.8793	0.7735	7.4846	0.614	115.0738
IFCNN [32]	0.9349	0.8305	0.6586	2.9408	0.8608	8.3031(2)	0.7341	40.6426
Proposed	**1.2559**	**0.8500**	**0.7444**	2.9653(4)	0.8939(4)	8.001	**0.8263**	38.9546

**Table 4:** Average experimental results of different MFIF methods on the MFFW dataset.

Methods	MFFW Dataset							
	Q_MI_	Q_NICE_	Q_G_	Q_M_	Q_P_	AG	Q_CB_	Q_CV_
NSCT-RR [12]	1.0951(3)	0.8363(3)	0.6723	2.9606(2)	0.7580	7.7225(4)	0.7335(4)	109.7688(4)
MWGF [22]	1.0355	0.8329	0.6855(4)	2.9433	**0.7848**	7.5424	0.7464(2)	404.5932
HMD [26]	1.1189(2)	0.8392(2)	0.6936(2)	2.9520(3)	0.7602(4)	7.7240(3)	0.7395	402.4889
GFDF [24]	1.0511(4)	0.8341(4)	0.6873(3)	**2.9621**	0.7778(2)	7.6493	0.7426(3)	104.9676(2)
YMY [16]	0.8589	0.8238	0.6168	2.9346	0.7110	7.0522	0.6704	123.4168
CSR [17]	0.7110	0.8181	0.5052	2.9142	0.6152	7.0623	0.5539	180.1241
ECNN [6]	0.7441	0.8192	0.4697	2.9198	0.5585	7.6325	0.6758	107.0475(3)
MFF-SSIM [5]	0.8266	0.8225	0.5688	2.9441	0.6868	7.8036(2)	0.7099	**104.8533**
MFF-GAN [4]	0.7043	0.8174	0.3973	2.8535	0.4950	**9.0122**	0.5616	239.3639
U2Fusion [31]	0.7258	0.8183	0.4754	2.8684	0.5743	7.1704	0.5764	192.0370
IFCNN [32]	0.7811	0.8204	0.5170	2.9179	0.6292	7.7187	0.6362	123.2416
Proposed	**1.1316**	**0.8405**	**0.6990**	2.9485(4)	0.7620(3)	7.7067	**0.7481**	417.1856

## Data Availability

Data sharing not applicable.
